# The influence of gender on CD4^+^ Treg cell function in acute ischemic stroke prognosis

**DOI:** 10.3389/fneur.2025.1626494

**Published:** 2025-08-28

**Authors:** Hui Na, Yue Gu, Yang Liu, Shiliang Xia

**Affiliations:** ^1^Department of Neurology, Hulunbuir People's Hospital, Hulunbuir, China; ^2^Department of Geriatrics, Yantai Affiliated Hospital of Binzhou Medical University, Yantai, China; ^3^Department of Neurology, Minhang Hospital, Fudan University, Shanghai, China

**Keywords:** CD4^+^ Treg cells, acute ischemic stroke, gender differences, stroke prognosis, immune modulation

## Abstract

**Objective:**

This study aimed to evaluate the influence of gender on the prognostic value of CD4^+^ Treg cells in patients with acute ischemic stroke.

**Methods:**

A prospective cohort study was conducted at Minhang Hospital, enrolling 225 patients with acute ischemic stroke. CD4^+^ Treg cell counts were measured by flow cytometry within 24 h of admission, and stroke prognosis was assessed at 3 months using the mRS. Univariate and multivariable logistic regression models were used to identify prognostic factors, and an interaction analysis was conducted to examine whether gender moderated the effect of Treg cell levels on outcomes.

**Results:**

Multivariable analysis revealed that infarct volume (OR = 1.08, 95% CI: 1.03–1.13, *p* = 0.0028), NIHSS score (OR = 1.30, 95% CI: 1.17–1.45, *p* < 0.0001), and WBC count (OR = 1.32, 95%CI: 1.05–1.67, *p* = 0.0172) were independent predictors of stroke prognosis. Higher CD4^+^ Treg cell counts were significantly associated with better prognosis in male patients (OR = 0.995, 95% CI: 0.992–0.999, *p* = 0.008), but showed no significant association in female patients (OR = 0.999, 95%CI: 0.998–1.001, *p* = 0.826). The interaction analysis confirmed that gender significantly moderated the relationship between CD4^+^ Treg cell counts and stroke prognosis (*p* = 0.0198). Additionally, segmented regression analysis revealed a nonlinear association between Treg cell counts and stroke prognosis in male patients, with specific thresholds indicating variable effects on prognosis.

**Conclusion:**

Gender plays a critical role in modulating the immunoregulatory effects of CD4^+^ Treg cells on stroke prognosis, with male patients deriving significant benefit from higher Treg cell counts.

## 1 Introduction

Stroke remains a leading cause of mortality and disability worldwide, with ischemic stroke accounting for the majority of cases (1). Despite advances in acute stroke treatments such as thrombolysis and mechanical thrombectomy, substantial heterogeneity persists in long-term outcomes, with immune responses playing a key role (2). Regulatory T cells (Tregs), particularly the CD4 + subset, play a pivotal role in suppressing inflammation and promoting neurorepair after stroke (3, 4). Immunomodulation mediated by CD4^+^ Treg cells can attenuate excessive immune activation and subsequent central nervous system (CNS) damage by inducing anti-inflammatory cytokines such as interleukin-10 (IL-10) and transforming growth factor-beta (TGF-*β*), thereby facilitating functional recovery (5–7).

Gender differences not only influence stroke incidence and prognosis, but also modulate immune responses through T cell activity (8). Treg cell function is influenced by sex hormones such as estrogen and testosterone, with accumulating evidence implicating their roles in post-stroke inflammation (9). In addition, emerging studies suggest that genetic polymorphisms (e.g., FOXP3), gut microbiota composition, and lifestyle factors (e.g., stress, physical activity) may serve as sex-specific modulators of Treg-mediated immune responses (10, 11). Furthermore, the immunological effects of sex hormones appear to vary before and after menopause in women, with estrogen-driven Treg expansion predominantly occurring in the premenopausal phase (12, 13). However, the specific influence of gender on CD4 + Treg cell function and its relationship with stroke prognosis remains poorly understood. This study aims to investigate whether patient gender influences the modulatory role of CD4 + Treg cells in stroke prognosis, thereby providing insights into personalized immunotherapeutic strategies for post-stroke recovery.

## 2 Methods

### 2.1 Study design

**Figure 1:**
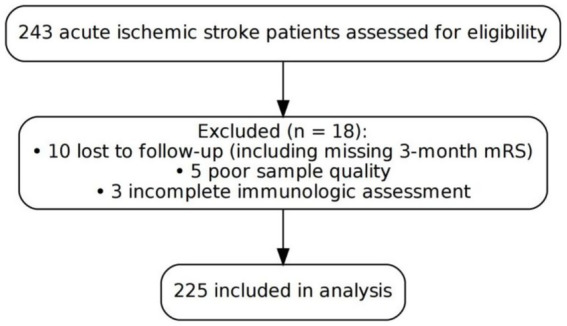
Patient flowchart of enrollment, exclusion, and inclusion for final analysis.

A single-center prospective cohort study was conducted at the Department of Neurology, Minhang Hospital, Fudan University, enrolling consecutive patients with acute ischemic stroke between January 2022 and December 2023. A total of 243 patients diagnosed with acute ischemic stroke were assessed for eligibility. Among them, 18 patients were excluded, including 10 lost to follow-up [e.g., missing 3-month modified Rankin Scale (mRS) data], 5 due to poor sample quality, and 3 due to incomplete immunologic assessment. A total of 225 patients were ultimately included in the analysis. All included participants met the eligibility criteria and completed both the 3-month follow-up and immunological testing (Figure 1).

The clinical study was approved by the ethical review board of Minhang Hospital, Fudan University, Shanghai, China. All procedures were conducted in accordance with the ethical standards of the Declaration of Helsinki. Written informed consent was obtained from all participants or their legally authorized representatives.

### 2.2 Participants in the study

Inclusion criteria: (1) Patients aged ≥18 years, diagnosed with acute ischemic stroke according to International Stroke Association guidelines, confirmed by imaging (MRI or CT) (14); (2) Time from symptom onset to hospital admission ≤24 h (3) Patients with complete baseline data including clinical history, immune markers, and infarct volume measurement. (4) Patients with a pre-stroke mRS score<2, to ensure that baseline functional independence was preserved prior to the index stroke event.

Exclusion criteria were as follows: (1) A history of ischemic or hemorrhagic stroke, traumatic brain injury, or other central nervous system diseases likely to cause permanent neurological deficits; (2) Severe organ failure (e.g., cardiac, hepatic, or renal) or active systemic infection; (3) Inability to provide informed consent or to complete baseline clinical and laboratory assessments; (4) Loss to follow-up or failure to complete 3-month mRS evaluation.

### 2.3 Data acquisition

#### 2.3.1 Clinical information

Demographic data (age, gender, body mass index [BMI], stroke history, smoking, and alcohol use) and clinical data (stroke subtype based on the Trial of Org 10,172 in Acute Stroke Treatment [TOAST] classification, and the presence of cardiovascular comorbidities such as hypertension, diabetes mellitus, dyslipidemia, or atrial fibrillation) were collected. The time of stroke onset was also recorded. Infarct volume on T2-weighted MRI was measured in cubic centimeters (cm^3^) using 3D reconstruction software.

#### 2.3.2 Laboratory examination

Immune markers: Peripheral venous blood (5 mL) was collected from patients within 24 h of admission using ethylenediaminetetraacetic acid (EDTA) anticoagulant tubes. Mononuclear cells were isolated by Ficoll density gradient centrifugation. The cells were resuspended in phosphate-buffered saline (PBS) containing 2% fetal bovine serum and stained with the following fluorochrome-conjugated monoclonal antibodies: CD3-fluorescein isothiocyanate (CD3-FITC), CD4-peridinin chlorophyll protein (CD4-PerCP), CD25-allophycocyanin (CD25-APC), and CD127-phycoerythrin (CD127-PE) (BD Biosciences, USA). Staining was performed at 4°C in the dark for 30 min. After washing, the cells were fixed in 1% paraformaldehyde and analyzed using a BD FACSCanto II flow cytometer. FlowJo software (version 10.6.2) was used for data analysis. TTreg cells were defined as CD3^+^CD4^+^CD25^high^CD127^low^. At least 50,000 CD3^+^ lymphocyte events were recorded per sample (Supplementary Figure 1).

Laboratory measurements: Blood samples were collected in the morning after an 8-h fast to assess biochemical markers, including hemoglobin concentration, platelet count, low-density lipoprotein (LDL), homocysteine, fasting blood glucose, and uric acid, using standard clinical chemistry methods.

#### 2.3.3 Prognostic evaluation

mRS was used during telephone or clinic follow-up at 3 months to evaluate functional recovery after stroke. Favorable prognosis was defined as mRS score 0–2, and unfavorable prognosis was defined as mRS score 3–6.

### 2.4 Statistical evaluation

All statistical analyses were performed using R statistical software (version 4.2.1). For univariate comparisons, the Shapiro–Wilk test was used to assess the normality of continuous variables, and Levene’s test was applied to evaluate the homogeneity of variances. Based on these results, either the independent t-test or the Mann–Whitney U test was used for continuous variables, and the chi-square test was used for categorical variables. Multivariable analyses were performed using multivariable logistic regression, and interaction terms were incorporated into the models to test for moderation effects. Segmented regression analysis was conducted using the “segmented” package in R. Inflection points (breakpoints) were automatically identified by the maximum likelihood estimation algorithm, and separate regression coefficients were calculated for each interval. Statistical significance was defined as *p* < 0.05.

## 3 Results

### 3.1 Baseline characteristics

A total of 225 patients with acute ischemic stroke were included in the final analysis. Based on the 3-month mRS scores, 167 patients were classified as having a favorable prognosis (mRS 0–2), and 58 as having an unfavorable prognosis (mRS 3–6). There were no differences between groups in terms of age (66.07 ± 13.75 years vs. 69.31 ± 15.35 years, *p* = 0.078) and sex distribution (males, 61.45% vs. 60.34%, *p* = 0.882). Smoking status and infarct volume differed significantly between the two groups (*p* = 0.021 and *p* < 0.001, respectively), with the favorable prognosis group having a significantly smaller infarct volume (2.53 ± 5.03 vs. 13.43 ± 43.06 cm^3^, *p* < 0.001). On admission, the National Institutes of Health Stroke Scale (NIHSS) scores were statistically lower in the favorable outcome group (3.02 ± 2.75 vs. 6.40 ± 4.84, *p* < 0.001). The number of CD4^+^ Treg cells significantly increased in patients with a favorable prognosis (501.77 ± 187.24 vs. 450.90 ± 201.55, *p* = 0.033), and WBC counts decreased (7.39 ± 1.45 vs.7.95 ± 1.56, *p* = 0.013) (Table 1).

**Table 1:** Baseline demographic and clinical characteristics.

Characteristics	Favorable prognoses group (*n* = 167)	Unfavorable prognoses group (*n* = 58)	*p*-value
Age (years)	66.07 ± 13.75	69.31 ± 15.35	0.078
Gender (Male), *n* (%)	102 (61.45)	35 (60.34)	0.882
Smoking, *n* (%)	38 (22.75)	5 (8.77)	0.021
Alcohol consumption, *n* (%)	21 (12.57)	4 (7.02)	0.25
Systolic blood pressure on admission (mmHg)	138.86 ± 18.54	143.48 ± 21.35	0.12
Diastolic blood pressure on admission (mmHg)	81.34 ± 10.15	82.16 ± 11.24	0.621
Heart rate (bpm)	76.17 ± 8.33	79.48 ± 12.96	0.123
Infarct volume (cm^3^)	2.53 ± 5.03	13.43 ± 43.06	<0.001
Hypertension, *n* (%)	111 (66.47)	44 (75.86)	0.183
Diabetes, *n* (%)	54 (32.34)	18 (31.03)	0.855
Lipid metabolism disorders, *n* (%)	24 (14.37)	9 (15.52)	0.832
Atrial fibrillation, *n* (%)	13 (7.78)	9 (15.52)	0.088
Personal history of stroke, *n* (%)	10 (5.99)	14 (24.14)	<0.001
Family history of stroke, *n* (%)	7 (4.19)	0 (0.00)	0.195
NIHSS Score on Admission	3.02 ± 2.75	6.40 ± 4.84	<0.001

### 3.2 Multivariable logistic regression results

In the multivariable logistic regression analysis, we adjusted for the following confounders: age, gender, hypertension, diabetes mellitus, dyslipidemia, atrial fibrillation, prior stroke history, smoking, and alcohol intake. The analysis demonstrated a significant correlation between infarct volume and poor prognosis (OR = 1.08, 95%CI = 1.03–1.13, *p* = 0.0028), indicating that each 1cm^3^ increment in infarct volume corresponds to an 8% elevation in the risk of unfavorable prognosis. NIHSS score on admission had a strong correlation with prognosis (OR = 1.30, 95%CI = 1.17–1.45, *p* < 0.0001), indicating that each 1-point increment in NIHSS score corresponds to a 30% elevation in the chance of unfavorable prognosis. The white blood cell (WBC) count was an independent and significant predictor in the adjusted model (OR = 1.32, 95%CI = 1.05–1.67, *p* = 0.0172), with a one-unit increase corresponding to a 32% increase in the odds for poor prognosis. The influence of CD4^+^ Treg cells neared significance (OR = 1.00, 95%CI = 1.00–1.00, *p* = 0.0774) (Table 2).

**Table 2:** Stroke subtype classification based on TOAST criteria.

Characteristics	Favorable prognoses group (*n* = 167)	Unfavorable prognoses group (*n* = 58)	*p*-value
TOAST classification, *n* (%)			0.41
CE	4 (2.40)	1 (1.72)	
CE	0 (0.00)	1 (1.72)	
LAA	97 (58.08)	39 (67.24)	
SAA	42 (25.15)	12 (20.69)	
SOE	4 (2.40)	1 (1.72)	
SUE	20 (11.98)	4 (6.90)	

### 3.3 Gender as a moderator of CD4^+^ Treg cell effect on stroke outcome

Interaction analysis revealed a significant gender-specific effect of CD4^+^ Treg cell counts on stroke prognosis (interaction *p* = 0.0198). In male patients, CD4 + Treg cells had a significant negative correlation with poor prognosis (adjusted OR = 0.995, 95%CI = 0.992–0.999, *p* = 0.008), indicating that each unit increase in CD4^+^ Treg cells diminishes the chance of poor prognosis by approximately 0.5%. No significant correlation between CD4^+^ Treg cells and prognosis was found in female patients (adjusted OR = 0.999, 95%CI = 0.998–1.001, *p* = 0.826) (Table 3).

**Table 3:** Laboratory and hematological parameters on admission.

Characteristics	Favorable prognoses group (*n* = 167)	Unfavorable prognoses group (*n* = 58)	*p*-value
Lymphocyte count (cells/μL)	20370.35 ± 4224.76	20089.60 ± 5025.57	0.278
CD19 B cells (cells/μL)	2331.04 ± 1183.30	2378.29 ± 1338.87	0.879
Breg cells (cells/μL)	557.57 ± 358.44	609.26 ± 419.28	0.693
CD3 CD4 T cells (cells/μL)	8183.84 ± 2773.55	7658.72 ± 2998.53	0.259
CD4 Treg cells (cells/μL)	501.77 ± 187.24	450.90 ± 201.55	0.033
CD3 CD45RA T cells (cells/μL)	5321.05 ± 2045.00	5126.14 ± 2048.80	0.879
CD3 CD45RA CD8 T cells (cells/μL)	985.42 ± 617.28	981.10 ± 576.03	0.778
CD8 Treg cells (cells/μL)	301.27 ± 187.32	324.60 ± 173.00	0.168
CD3 T cells (cells/μL)	13556.01 ± 3556.01	13033.52 ± 4190.12	0.379
DNT CD3 CD4 CD8 T cells (cells/μL)	583.70 ± 349.99	556.66 ± 337.82	0.695
CD3 CD4 CD8 T CELL	5149.61 ± 1968.04	5440.41 ± 2072.41	0.436
CD3 CD4 CD8 T CELL	7135.61 ± 2438.02	6644.54 ± 2556.85	0.331
RBC Count (×10^12/L)	4.34 ± 0.43	4.31 ± 0.47	0.757
WBC Count (×10^9/L)	7.39 ± 1.45	7.95 ± 1.56	0.013
Hemoglobin (g/L)	137.81 ± 11.62	138.12 ± 14.74	0.604
Platelet count (×10^9/L)	214.30 ± 44.81	210.16 ± 49.58	0.196
LDL (mmol/L)	2.61 ± 0.71	2.73 ± 0.83	0.399
Homocysteine (μmol/L)	12.75 ± 2.32	13.06 ± 2.43	0.135
Fasting blood glucose (mmol/L)	5.75 ± 0.89	5.88 ± 0.98	0.436
Uric acid (μmol/L)	303.15 ± 67.40	283.21 ± 74.73	0.082

Tables 1–3: continuous variables are expressed as mean ± SD and compared using *t*-tests or Mann–Whitney *U* tests as appropriate. Categorical variables are presented as n (%) and compared using chi-square tests. Abbreviations: CE, cardioembolism; LAA, large artery atherosclerosis; SAA, small artery arteriolosclerosis; SOE, stroke of other determined etiology; SUE, stroke of undetermined etiology; NIHSS, National Institutes of Health Stroke Scale; RBC, red blood cells; WBC, white blood cells; LDL, low-density lipoprotein.

**Figure 2:**
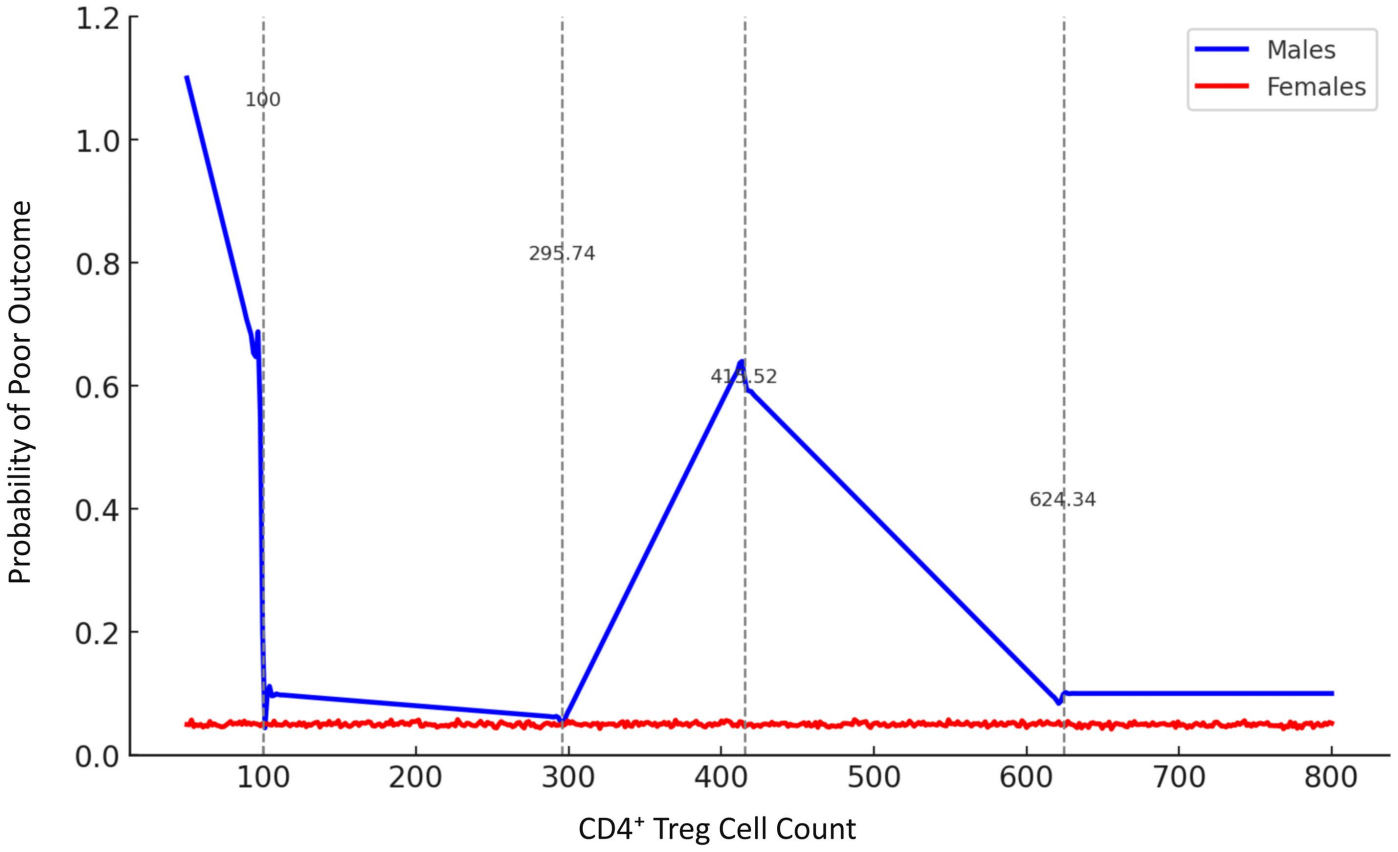
Gender-stratified smooth curve analysis of the association between CD4^+^ Treg cell count and stroke prognosis. Smooth curve fitting was performed using a generalized additive model (GAM), adjusting for age, infarct volume, NIHSS score, and other confounders. The blue line represents male patients, and the red line represents female patients. Vertical dashed lines indicate inflection points estimated by penalized spline regression at 100, 295.74, 415.52, and 624.34 cells/μL. In males, the relationship between CD4^+^ Treg cell count and poor prognosis probability was non-linear, while females showed no significant trend.

Analysis of smooth curves in male patients revealed a variable correlation between CD4^+^ Treg cells and prognosis. In male patients, CD4^+^ Treg cell counts below 100 were associated with a higher likelihood of poor prognosis. The risk decreased significantly (p-trend<0.001) as long as the amount of CD4^+^ Treg cells was up to or above 295.74 Following additional increases to 415.52, the danger increased once more, followed by a decrease at 624.34, when the risk stabilized at low levels (Figure 2).

### 3.4 Segmented regression analysis of CD4^+^ Treg cell count and the stroke prognosis

**Figure 3:**
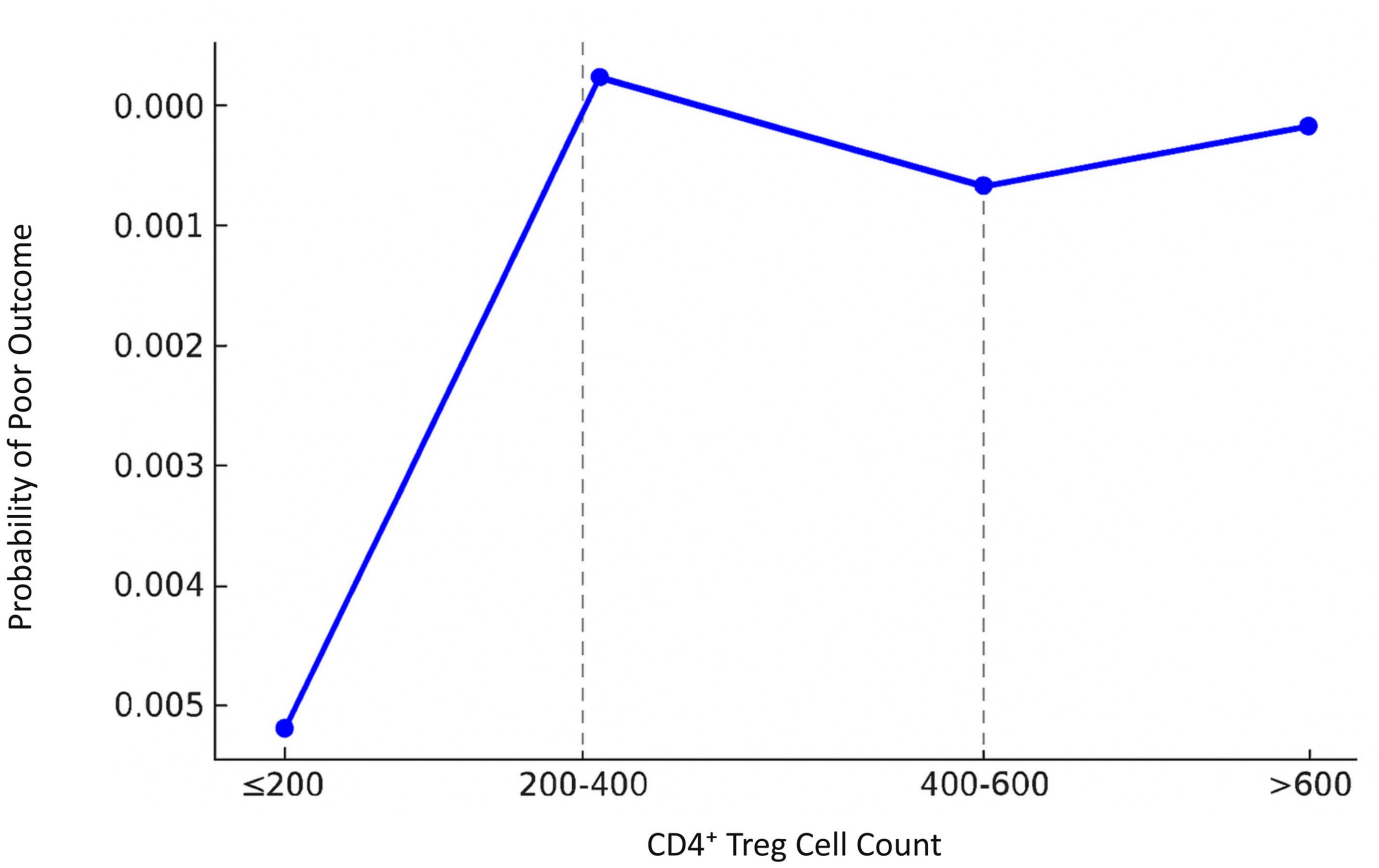
Segmented regression analysis of CD4^+^ Treg cell count and stroke prognosis in male patients. A segmented linear regression model was applied to assess the effect of CD4^+^ Treg count on the probability of poor outcome across four intervals (≤200, 200–400, 400–600, >600 cells/μL). Regression coefficients were estimated for each segment. Thresholds were determined using the segmented R package with maximum likelihood estimation. The model revealed significant heterogeneity in prognosis association across CD4^+^ Treg levels.

Segmented regression analysis demonstrated that the effect of CD4^+^ Treg cells on the prognosis of stroke has significant heterogeneities over a number of thresholds. For the low range (CD4^+^ Treg ≤200), the count of CD4^+^ Treg cells showed a positive correlation with unfavorable prognosis and it exhibited an regression coefficient of 0.0017, *p* = 0.094. In the intermediate range (200 < CD4^+^ Treg ≤400), the regression coefficient was 0.0003, *p* = 0.606. In the elevated range (400 < CD4^+^ Treg ≤600), the regression coefficient was −0.0002, *p* = 0.700. In the most upper range (>600), the regression coefficient was −8.99e-06, *p* = 0.977 (Figure 3 and Tables 4, 5).

**Table 4:** Multivariate logistic regression analysis of prognostic factors in patients with acute ischemic stroke.

Characteristics	OR (95% CI)	*p*-value
Infarct volume (cm^3^)	1.08 (1.03, 1.13)	0.0028
NIHSS Score	1.30 (1.17, 1.45)	<0.0001
CD4 + Treg Cells (cells/μL)	1.00 (1.00, 1.00)	0.0774
White blood cell count (×10^9^/L)	1.32 (1.05, 1.67)	0.0172

**Table 5:** Interaction analysis of the Effect of CD4 + Treg cells on stroke prognosis by gender.

Gender	OR (95% CI)	*p*-value	Interaction *p*-value
Male	0.995 (0.992, 0.999)	0.008	0.0198
Female	0.999 (0.998, 1.001)	0.826	

## 4 Discussion

The present study explored the association of peripheral CD4^+^ Treg cells and prognosis in patients with acute ischemic stroke, and identified that higher circulating CD4^+^ Treg counts were associated with a better post-stroke prognosis. And importantly, gender was revealed as the significant moderator of the effect of CD4^+^ Treg cells. Exerting a more significant impact on prognosis in male patients, whilst no such effect was noted in females.

Our data confirm the essential role of CD4^+^ Treg cells in controlling immunological response post-stroke. Earlier studies support this premise. Cadavid M et al. demonstrated reduced neuroinflammation and improved stroke recovery after CD4^+^ Treg cell expansion in subjects experiencing acute ischemic stroke. Their later research revealed that increased numerical Treg cells were accompanied with improved neurological function at the time point of 90 days post-stroke (5). Li et al. also found a link between increased CD4^+^ Treg cell numbers and favorable 30-day post-stroke outcomes (15). The results are consistent with studies in our laboratory that suggest Treg cells suppress CNS injury by suppressing effector T cells and inflammatory mediators, thus promoting functional recovery (16–18).

The gender-specific regulation of Treg cell function distinguishes this study. In male patients, elevated CD4^+^ Treg cells were substantially correlated with improved prognosis (19). Yan et al. (20) found that male stroke patients with elevated testosterone levels exhibited significantly greater Treg cell numbers and improved neurological recovery compared to those with lower testosterone levels. With respect to male patients, testosterone levels and Treg cell counts showed a significant positive connection. Regression analysis revealed that higher testosterone levels enhanced Treg-mediated control of pro-inflammatory cytokines, including interleukin-6 (IL-6) and tumor necrosis factor-alpha (TNF-*α*) (21). Guo and Yang et al. similarly found that testosterone not only raised Treg cell counts but also enhanced their suppressive capacity by TGF-*β*, hence reducing post-stroke brain damage (22, 23). The results suggest that testosterone may increase Treg-mediated immunological regulation, hence reducing neuroinflammation in male patients and improving stroke outcomes (18, 24).

In contrast, the relationship between CD4^+^ Treg cell counts and stroke prognosis in female patients is more complex. Although some studies have demonstrated a positive role of estrogen in enhancing Treg cell function, the results of the present study differed and failed to observe a significant correlation between the number of Treg cells and prognosis in female patients (25, 26). Animal studies by Ahnstedt et al. (27) found lower levels of Treg cell infiltration in female mice after stroke, and in the chronic phase of the disease Treg cells showed a different response to neurological inflammation was weakly regulated, which differed from immunoregulation during recovery in male mice. The lack of significant correlation between Treg cells and prognosis with the findings observed in female patients in this study is informative.

More importantly, a nonlinear relationship between CD4^+^ Treg cells and prognosis was identified via smooth curve methodology analysis as well as segmented regression in male patients. At low Treg levels (<100), the probability of poor prognosis was elevated; however, when Treg cell levels rose to roughly 295, the risk diminished. Subsequent increases to approximately 415 prompted a revival in risk; however, the risk subsequently diminished and stabilized at elevated levels (>624). This finding might suggest the dual role of Treg cells in post-stroke recovery. At low levels, there may be not enough Treg cells to regulate inflammation effectively, so this could cause stroke outcomes to become worse. Previous research showed that Treg cells could protect the CNS in the acute post-stroke phase by inhibiting inflammatory responses and reducing pro-inflammatory cytokines secretion, such as interleukin-17 (IL-17) and interferon-gamma (IFN-*γ*) (28–30). When the numbers of Treg cells are elevated to a certain threshold, they can carry out improved immunosuppression and consequent induction of neurorepair. This excessiveness in Treg cell numbers could in turn result in over-suppression of beneficial immune responses required for tissue clearance and repair (31–33). Shi et al. (34) suggested that over-inhibition of immune responses could be deleterious for neuroregeneration, translating into poor outcomes. Following the proliferation of Treg cells, immunological balance might be reconstituted and neural repair apparatus replenished. This discovery adds a new dimension to how Treg cells modulate post-stroke immune responses, offering us an important opportunity to understand the precise role in details. Future studies need to determine the optimal level of Treg cells in post-stroke repair, allowing for personalized regulation to achieve better stroke outcomes.

This study highlights the importance of CD4^+^ Treg cells in post-stroke immune modulation and the impact of gender on the effect of these cells on stroke outcome. Increases in Treg cell numbers contributed to significantly improved prognosis in male patients, but not female patients. It is likely that sex hormones modulate the efficacy of Treg cells by complex signaling pathways resulting in impact on stroke recovery. In addition, smoking status differed significantly between the prognosis groups at baseline, although it was not included in the main analysis. As a well-known vascular risk factor, smoking may influence stroke outcomes through inflammatory or endothelial mechanisms. This observation deserves attention in future investigations. In the future, large scale multicenter trials are needed to confirm these results and provide a stronger theoretical framework for individualized treatment of stroke.

This study has several limitations. First, it was conducted at a single center with a relatively limited sample size, especially in the female subgroup, which may restrict the generalizability of the findings. Second, sex hormone levels such as estrogen and testosterone were not directly measured, limiting mechanistic interpretation of the gender-specific effects observed. Third, although smoking status differed between prognosis groups, it was not included in the main regression models and should be explored further in future studies. Lastly, the observational nature of this study precludes causal inference, and no experimental validation was performed. These limitations should be addressed in future prospective and mechanistic studies.

## Data Availability

The original contributions presented in the study are included in the article/Supplementary material, further inquiries can be directed to the corresponding author/s.
