# Supplementary figures and images for: The influence of gender on CD4+ Treg cell function in acute ischemic stroke prognosis
Source: Front Neurol. 2025 Aug 28;16:1626494. doi: 10.3389/fneur.2025.1626494 (PMC12422904; doi:10.3389/fneur.2025.1626494)

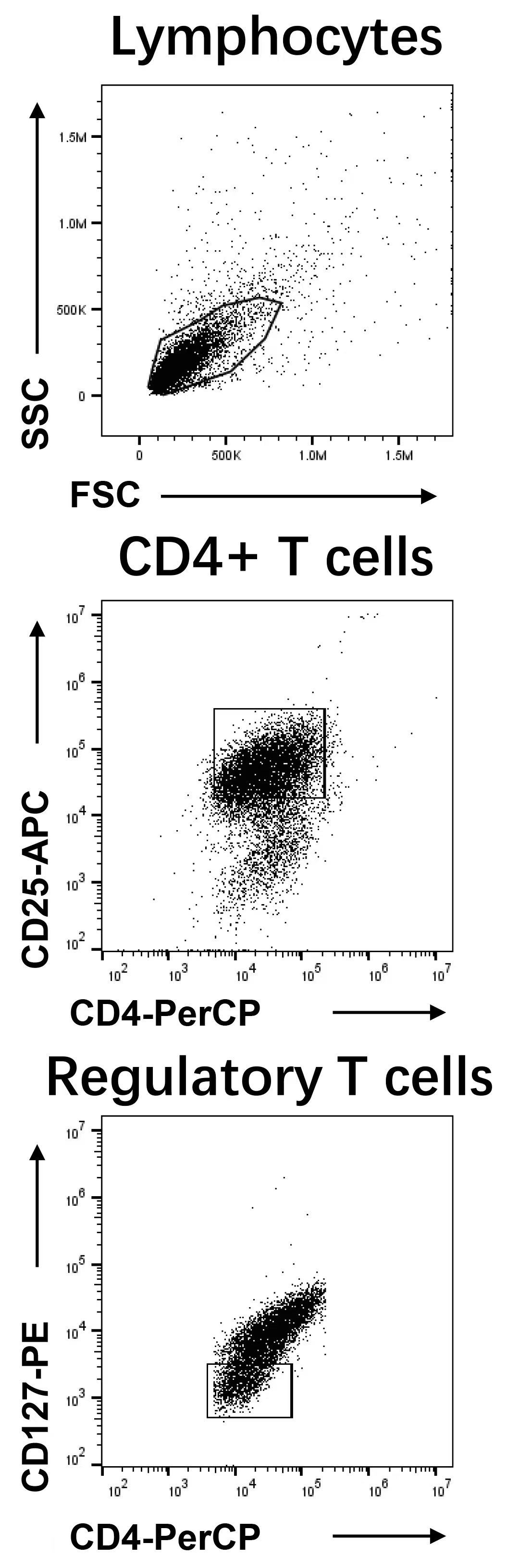

Supplement: Supplementary file 1 [file Image_1.jpeg]
